# QT Interval Evaluation in Right Ventricular Pacing: Validation of a Novel Formula

**DOI:** 10.1111/pace.70239

**Published:** 2026-04-03

**Authors:** Amr Abdin, Alhasan Almasri, Ibrahim Antoun, Charles Gieres, Christian Werner, Yvonne Bewarder, Christian Ukena, Igor Schwantke, Michael Böhm, Saarraaken Kulenthiran

**Affiliations:** ^1^ Department of Internal Medicine III, Cardiology, Angiology Intensive Care Medicine Saarland University Saarland University Medical Center Homburg/Saar Germany; ^2^ Department of Cardiology University Hospitals of Leicester NHS Trust Glenfield Hospital Leicester UK; ^3^ Department of Cardiovascular Sciences Clinical Science Wing University of Leicester Glenfield Hospital Leicester UK; ^4^ Department of Cardiology Hôpitaux Robert Schuman Luxembourg Luxembourg; ^5^ Ruhr‐University of Bochum Medical Department II Marien Hospital Herne Herne Germany; ^6^ Homburg Institute for CardioRenalMetabolic Medicine Universitätsklinikum Des Saarlandes Homburg/Saar Germany

**Keywords:** electrocardiogram, formula, pacemaker, QRS, QT interval

## Abstract

**Background:**

QT interval measurement in the presence of right ventricular pacing (RVP) represents a clinical challenge. We therefore aimed to derive and validate a formula for QT estimation during RVP in a large cohort of pacemaker patients.

**Methods and Results:**

We prospectively enrolled 100 patients in a derivation cohort and 487 in a validation cohort. Eligible patients had implanted pacemakers or implantable cardioverter‐defibrillators, preserved atrioventricular conduction, and a narrow native QRS. Standardized 12‐lead ECGs were recorded during intrinsic rhythm, atrial pacing (AP), and RVP at matched heart rates. Linear regression of QT during non‐paced rhythm against paced QT yielded the equation: **QT_non‐paced = 139.23 + 0.55 × QT_paced**. In the validation cohort (mean age 71 ± 13 years, 72% male), application of the formula showed a significant correlation between predicted and observed QT values (*r* = 0.50, *p* < 0.001). Bland–Altman analysis demonstrated a modest mean overestimation of 15 ms, with consistent accuracy across subgroups defined by sex, device type, and lead position. Compared directly with the Bogossian method, our formula showed superior performance, with a lower mean absolute deviation (26.2 vs. 39.9).

**Conclusion:**

This novel, heart rate‐independent formula provides a reliable method for estimating the intrinsic QT interval from paced measurements. It represents a practical tool to improve QT assessment and arrhythmic risk evaluation in patients with RVP, with potential implications for clinical decision‐making and drug safety monitoring.

## Graphical abstract

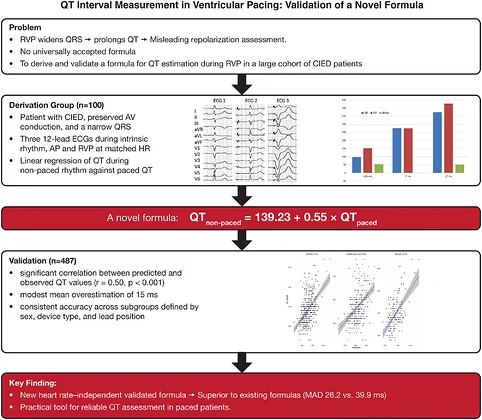

## 1 Introduction

Accurate measurement of the QT interval is essential for evaluating the risk of malignant arrhythmias and sudden cardiac death [1]. However, assessment of the QT interval in patients with right ventricular pacing (RVP) remains particularly challenging due to several electrophysiological factors [2, 3, 4, 5]. RVP leads to widening of the QRS complex, thereby artificially prolonging the QT interval by prolonging the depolarization phase [4, 6]. In addition, RVP alters the spatiotemporal pattern of ventricular activation, thereby modifying repolarization. These changes may result in a misleadingly prolonged QT interval that does not necessarily reflect the true duration of repolarization.

Over the past decades, different strategies have been proposed to adjust for this effect and to improve the accuracy of QT interval estimation during RVP. These approaches include: (i) subtraction of a fixed value from the measured QT interval [2, 3, 4], (ii) subtraction of a proportion of the paced QRS duration to account for pacing‐induced QRS prolongation [5, 7], and (iii) modification of QT measurement using specific adjustment formulas [8, 9]. Although each method attempts to mitigate the confounding influence of pacing, their applicability has been inconsistent, and none has been universally adopted in clinical practice. The presence of multiple competing formulas highlights the lack of a standardized approach while also emphasizing the importance of validating any correction method in independent patient populations beyond the original derivation cohorts.

In this context, the present study was designed with two main objectives. First, we aimed to compare QT intervals obtained during intrinsic rhythm and atrial pacing (AP) with those measured during RVP at matched heart rates in the same patients. Second, we sought to evaluate whether a newly derived correction formula could improve the accuracy of QT estimation in paced complexes. To achieve this, we validated the formula in a large independent cohort of patients with RVP.

## 2 Methods

### 2.1 Study Population

We included patients who attended the pacemaker outpatient clinic of Saarland University Medical Center between October 1, 2021, and January 31, 2022. The study protocol was approved by the local ethics committee (No. 327/21). Patients were enrolled consecutively during routine outpatient device follow‐up. To derive the correction formula, the first 100 eligible patients meeting the inclusion criteria were prospectively assigned to the derivation cohort. All subsequently enrolled eligible patients were assigned to an independent validation cohort. This allocation strategy was predefined before data analysis and was not based on randomization or post‐hoc selection. The separation into derivation and validation cohorts was chosen to avoid overfitting and to allow independent assessment of the formula's performance.

Inclusion criteria were as follows:

‐ Clinically stable patients without acute cardiac symptoms and hemodynamically stable.
‐ Patients with preserved atrioventricular (AV) conduction and implanted cardiac rhythm devices (dual‐chamber pacemakers or implantable cardioverter‐defibrillators [ICDs]) for standard clinical indications, primarily sinus node dysfunction or intermittent AV block for pacemakers, and primary or secondary prevention of sudden cardiac death for ICDs.
‐ Narrow native QRS (<120 ms).
‐ Ventricular rate between 50 and 155 bpm, consistent with the validated range of published heart rate correction formulas.

Exclusion criteria included:

‐ QRS duration >120 ms, indicating intraventricular conduction disturbance.
‐ Ventricular pacing dependency.
‐ Significant baseline ST–T abnormalities, which may confound repolarization assessment.

### 2.2 ECG Acquisition and Measurements

Twelve‐lead ECGs were digitally recorded using the SmartScript PC‐ECG system (Dr. Gerhard Schmidt GmbH) and verified by two certified cardiologists. Recordings were obtained at a paper speed of 50 mm/s and analyzed using digital calipers. For each patient in the derivation cohort, three ECGs were recorded: one at intrinsic rate, one during atrial pacing (AP), and one during RVP at the same rate as AP (80 or 90 bpm). The longest QT interval among the 12 leads was taken as the raw QT. Corrected QT (QTc) was calculated using Bazett's formula. AP ensured intrinsic AV conduction with a narrow QRS, allowing direct comparison of QT intervals measured under AP and VP at identical heart rates (Figure 1).

**FIGURE 1:**
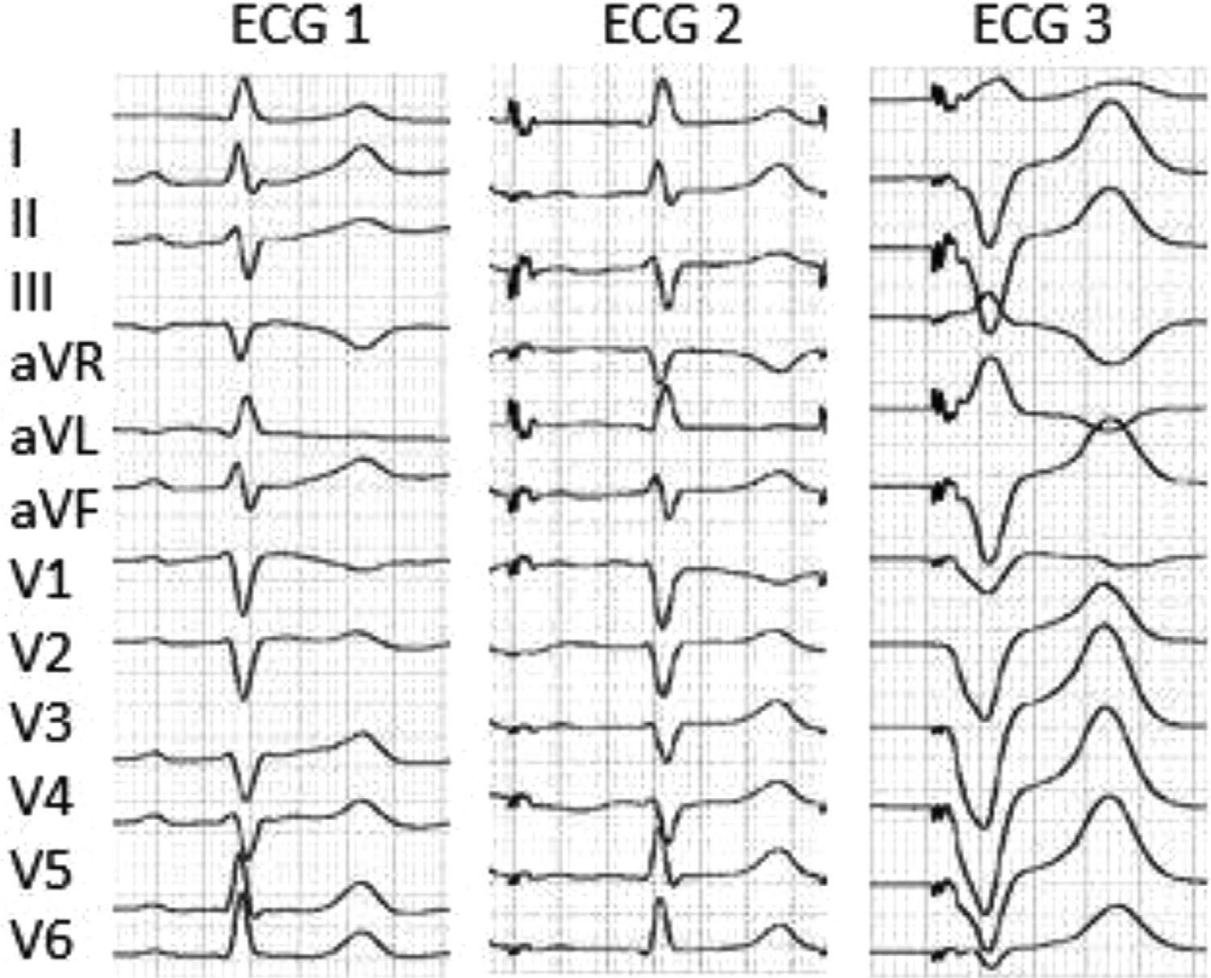
Example with three ECGs from the same patient. ECG1 shows the intrinsic rhythm, ECG2 shows atrial pacing, and ECG3 shows ventricular pacing. ECG2 and ECG3 were recorded at the same heart rate.

In the validation cohort, two ECGs per patient were obtained: one at intrinsic rhythm and one during VP at 80 bpm (*n* = 214, 44%) or 100 bpm (*n* = 273, 56%). All ECGs were independently analyzed by two certified cardiologists.

### 2.3 Statistical Analysis

Continuous variables are presented as mean ± standard deviation (SD). Data were entered into a dedicated electronic database (Microsoft Excel, SPSS, and R). Statistical significance was set at *p* < 0.05. Ordinary least squares linear regression was performed with QT during VP as the predictor and QT during non‐paced rhythm as the outcome. Regression coefficients (intercept b0, representing the QT value when QTpaced = 0, and slope b1, representing the change in QT per unit increase in QTpaced) were used to derive the formula: QTnon‐paced = b0 + (b1 × QTpaced). Correlation analyses were applied to assess the strength and significance of the association between predicted and observed values.

## 3 Results

### 3.1 Baseline Characteristics

The derivation cohort consisted of 100 patients with a mean age of 73 ± 11.4 years; 63% were male. The majority were pacemaker recipients (*n* = 84, 84%), while 16 patients (16%) carried an ICD (Table 1). The leading indication for pacemaker implantation was sinus node disease (*n* = 59, 59%), followed by AV node disease (*n* = 24, 24%). ICDs were implanted predominantly for secondary prevention of sudden cardiac death (*n* = 11, 11%) and in a smaller proportion for primary prevention (*n* = 6, 6%).

**TABLE 1:** Baseline characteristics of the derivation and validation groups. BMI: Body mass index; ICM: Ischemic cardiomyopathy; ICD: Implantable cardioverter defibrillator; LVEF: Left ventricular ejection fraction; PM: Pacemaker; RV: Right ventricle.

Baseline characteristics	Derivative group *n*: 100	Validation group *n*: 487
**Clinical**		
**Age ‐ years**	73,8 ± 11,4	71,3 ± 12,9
**Gender M: Male, F: Female no. (%)**	M: 63 (63), F: 37 (37)	M: 349 (72), F 138 (28)
**Height m**	1,71 ± 0,1	1,72 ± 0,09
**Weight kg**	79,7 ± 16,2	80,8 ± 18,1
**BMI, kg/m2**	27,2 ± 4,9	27,2 ± 5,0
**LVEF %**	53,5 ± 80	50,7 ± 11,4
**ICM — no. (%)**	13 (13)	108 (22)
**Coronary artery disease — no. (%)**	34 (34)	187 (38)
**Hypertension — no. (%)**	73 (73)	346 (71)
**Diabetes — no. (%)**	25 (25)	112 (23)
**History of atrial fibrillation or flutter — no. (%)**	56 (56)	204 (42)
QT (ms), intrinsic rhythm	381,5 ± 38,45	389,93 ± 34,3
QTc (ms), intrinsic rhythm	404,7 ± 35,9	411,29 ± 29,4
**Device characteristics**		
**Device 1: VVI‐PM, 2: DDD‐PM, 3: VVI‐ICD, 4: DDD‐ICD — no. (%)**	1: 1 (1), 2: 83 (83), 3: 3 (3), 4: 13 (13)	1: 23 (5), 2: 279 (57), 3: 156 (32), 4: 29 (6)
**Indication 1: SSS, 2: AV‐block, 3: primary prevention, 4: secondary prevention — no. (%)**	1: 59 (59), 2: 24 (24), 3: 6 (6), 4: 11 (11)	1: 193 (40), 2: 104 (21), 3: 76 (16), 4: 114 (23)
**RV lead position 1: apical, 2: septal — no. (%)**	apical 26 (26), septal 72 (72)	apical 225 (46), septal 260 (54)
**RV pacing %**	8,9 ± 17,1	9,13 ± 19,8
**Medication**		
**B‐Blocker — no. (%)**	74 (74)	379 (78)
**Calcium channel blocker — no. (%)**	24 (24)	116 (24)
**Amiodaron — no. (%)**	4 (4)	34 (7)
**Sotalol— no. (%)**	0 (0)	0
**Class I anti‐arrhythmic — no. (%)**	2 (2)	4 (1)
Other drugs cause QT prolongation — no. (%)	11 (11)	69 (14,2)

The validation cohort included 487 patients with a mean age of 71 ± 12.9 years; 72% were male (Table 1). Pacemaker recipients represented 62% (*n* = 302) of this group, while 38% (*n* = 185) had ICDs. As in the derivation cohort, sinus node disease was the most common pacemaker indication (*n* = 193, 40%), followed by AV node disease (*n* = 104, 21%). ICD indications included secondary prevention (*n* = 76, 16%) and primary prevention (*n* = 114, 23%). Septal RV lead positioning was more frequent in the derivation cohort (72%) than in the validation cohort (54%), reflecting institutional practice (septal positioning for pacemakers, apical for ICDs). The mean ventricular pacing percentage was similar in both cohorts (≈9%).

### 3.2 ECG Measurements

In the derivation cohort, AP was associated with mean QRS, JT, and QT values of 98.2 ± 10.3 ms, 276.9 ± 25.9 ms, and 375.0 ± 27.1 ms, respectively. During RVP, the corresponding values were QRS 152.4 ± 19.2 ms, JT 275.9 ± 26.5 ms, and QT 427.9 ± 30.8 ms (Figure 2). Thus, the QT interval was prolonged by ∆QT = 52.7 ms during VP compared to AP, which was largely attributable to QRS widening (∆QRS = 54.2 ms) (Table S1). The JT interval remained virtually unchanged (∆JT = −1 ms). The relative contribution of QRS duration to the QT interval increased from 26.2% during AP to 35.6% during VP, underscoring that pacing‐induced QT prolongation is primarily driven by QRS widening (Figure S1).

**FIGURE 2:**
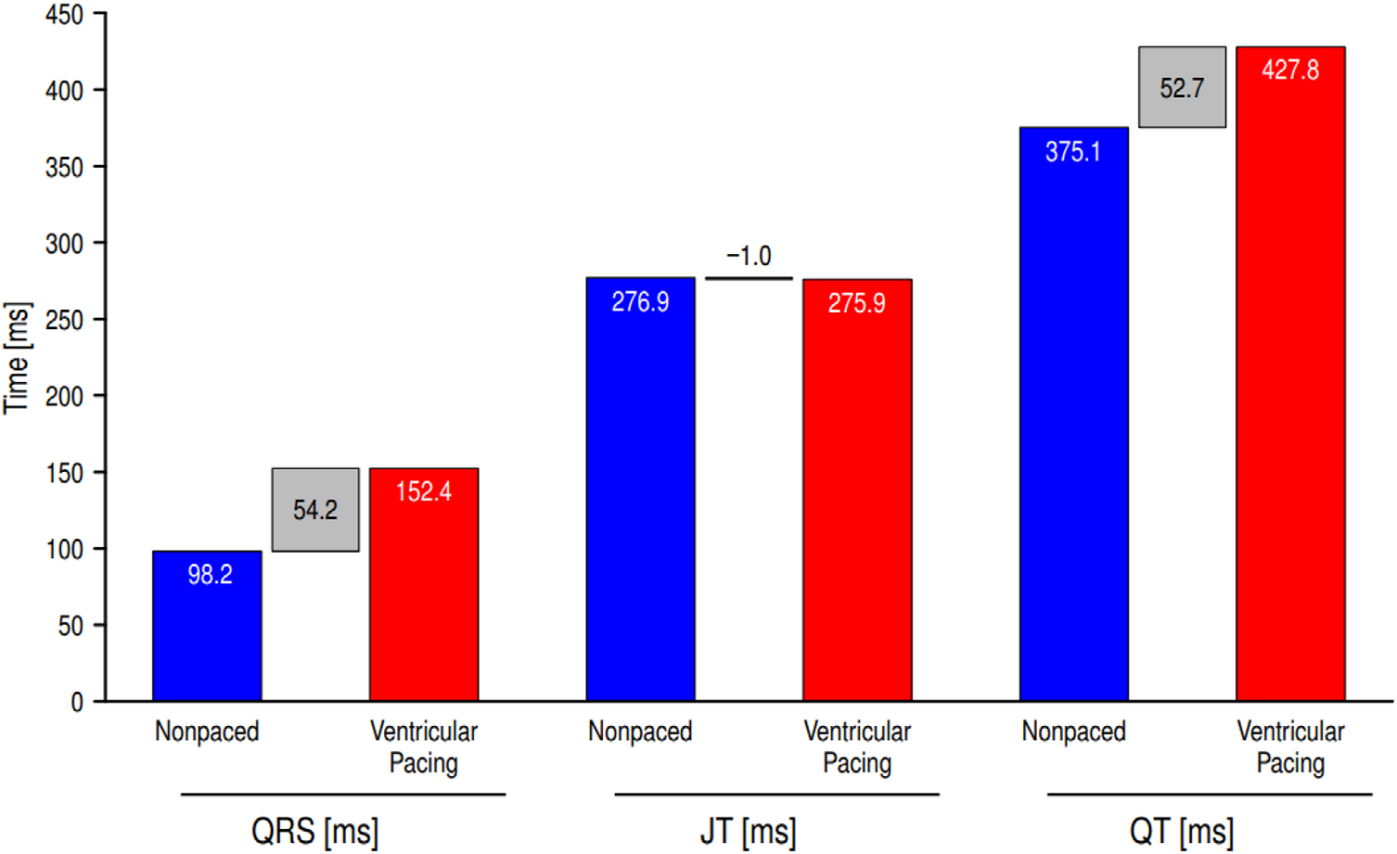
QRS, JT, and QT intervals in atrial pacing (AP) and ventricular pacing (VP), and their changes (Δ), in the derivative cohort. [Colour figure can be viewed at wileyonlinelibrary.com]

**Derivation of a correction formula**

In the derivation cohort, linear regression analysis with QTpaced as the predictor and QTnon‐paced as the outcome yielded the following equation:

**QTnon‐paced = 139.23 + 0.55 × QTpaced**.

QTpaced was a highly significant predictor (*p* < 0.001). Importantly, because all ECGs were recorded at matched heart rates, the formula is independent of heart rate. Rounding errors were negligible, with an average deviation of only −0.23 ms (Figure 3).

**FIGURE 3:**
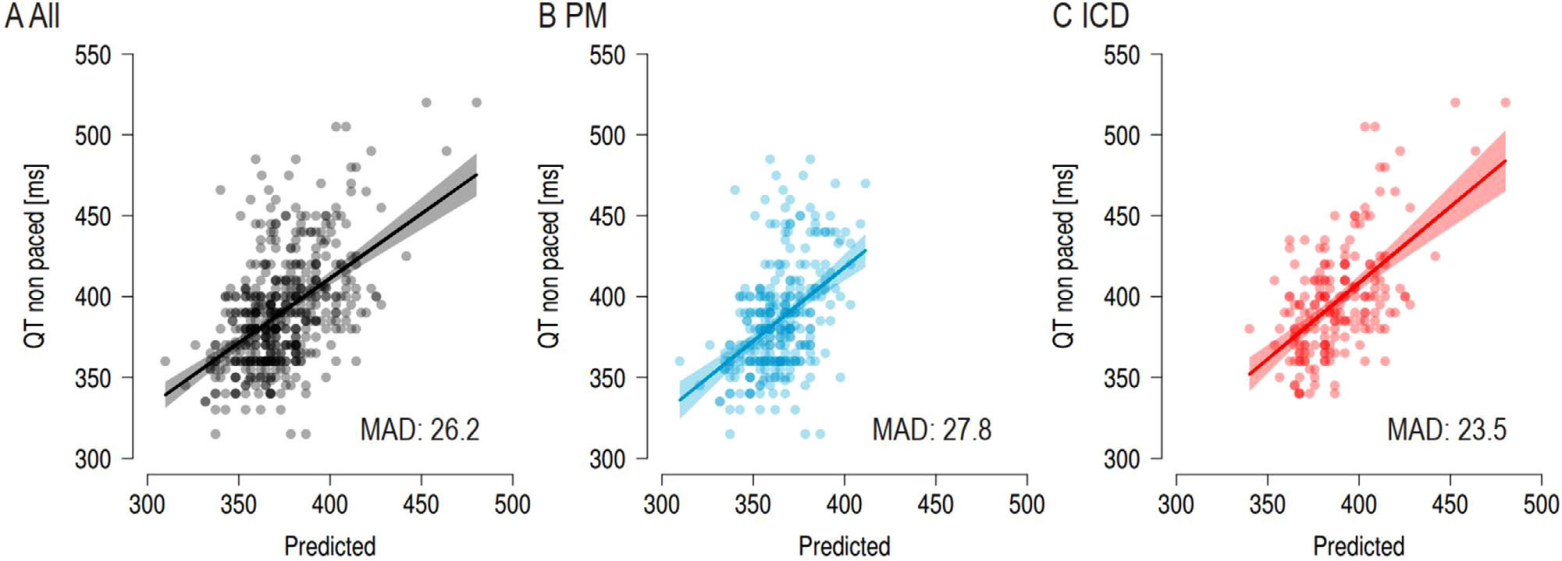
Forest plot illustrating the correlation coefficients between predicted and observed QT values. MAD = median absolute deviation, representing the average error (ms) between predicted and actual values. *X*‐axis: predicted values; *Y*‐axis: residuals (prediction − actual). Prediction accuracy (MAD, smaller = more accurate) is shown in parentheses next to the device label. [Colour figure can be viewed at wileyonlinelibrary.com]

### 3.3 Validation of the Formula

The formula was tested in the validation cohort of 487 patients. Patients were divided according to pacing rate: 214 (44%) at 80 bpm and 273 (56%) at 100 bpm. Similar to the derivation cohort, QT prolongation during VP could be attributed to QRS widening (Figure 4, Table S2). Subgroup analyses were performed by sex, device type (pacemaker vs. ICD), and lead position (septal vs. apical) (Table S3).

**FIGURE 4:**
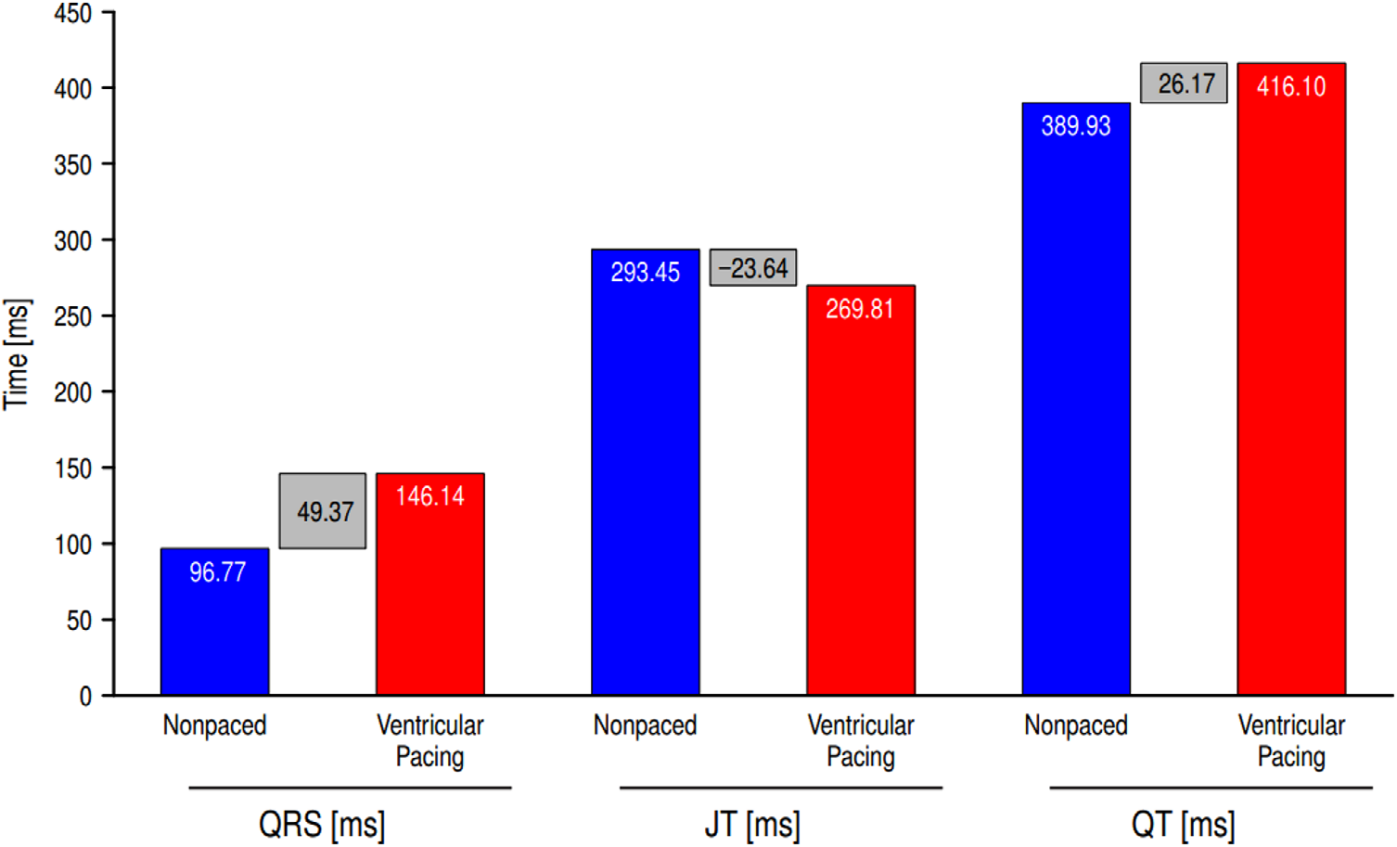
QRS, JT, and QT measurements in the non‐paced rhythm and during ventricular pacing at 80 or 100 bpm (validation cohort). [Colour figure can be viewed at wileyonlinelibrary.com]

In the overall validation population, the correlation between predicted and observed QT values was *r* = 0.50 (*p* ≤ 0.0001) (Table 2, Figure S2), confirming a statistically significant association. In all subgroup analyses, *r* exceeded 0.50, except in two groups with small sample sizes (apical pacemaker, *n* = 57; septal ICD, *n* = 17). A forest plot (Table 2, Figure S3) summarizes the subgroup results, with the overall correlation (*r* = 0.50) highlighted in red.

**TABLE 2:** Pearson correlation coefficients (*r*) with 95% confidence intervals and corresponding *p*‐values (for values <0.0001, *p* < 0.001) are shown for the total validation cohort and for subgroups according to gender, lead position, and device type. ICD: Implantable cardioverter defibrillator; PM: Pacemaker.

	All	PM	ICD
**All**	*r*=0.50, [95% CI: 0.43, 0.57], *p*<0.0001 (*n*=487)	*r*=0.45, [95% CI: 0.35, 0.53], *p*<0.0001 (*n*=302)	*r*=0.56, [95% CI: 0.46, 0.66], *p*<0.0001 (*n*=185)
**Male**	*r*=0.52, [95% CI: 0.44, 0.59], *p*<0.0001 (*n*=349)	*r*=0.47, [95% CI: 0.35, 0.57], *p*<0.0001 (*n*=199)	*r*=0.57, [95% CI: 0.45, 0.67], *p*<0.0001 (*n*=150)
**Female**	*r*=0.37, [95% CI: 0.22, 0.51], *p*<0.0001 (*n*=138)	*r*=0.35, [95% CI: 0.16, 0.51], *p*=0.0003 (*n*=103)	*r*=0.42, [95% CI: 0.10, 0.66], *p*=0.0127 (n=35)
**Apical**	*r*=0.55, [95% CI: 0.45, 0.63], *p*<0.0001 (*n*=225)	*r*=0.16, [95% CI: ‐0.11, 0.40], *p*=0.2464 (*n*=57)	*r*=0.59, [95% CI: 0.48, 0.68], *p*<0.0001 (*n*=168)
**Septal**	*r*=0.48, [95% CI: 0.38, 0.57], *p*<0.0001 (*n*=262)	*r*=0.51, [95% CI: 0.41, 0.59], *p*<0.0001 (*n*=245)	*r*=0.19, [95% CI: ‐0.32, 0.61], *p*=0.4747 (*n*=17)

Bland–Altman plots demonstrated a mean difference of −15.2 ms between predicted and observed QT values, corresponding to an average underestimation of approximately 15 ms. This bias was less pronounced in women and more marked in patients with septal RV lead positioning (Table S3).

## 4 Discussion

Because the QT interval encompasses both ventricular depolarization and repolarization, assessment of ventricular repolarization based solely on the QT interval in the presence of a wide QRS complex may be unreliable. We therefore developed a new formula to predict the non‐paced QT interval from the QT interval measured during RVP. This formula was validated in a large patient cohort and demonstrated high correlation and accuracy.

Widening of the QRS complex, as occurs with complete left bundle branch block or RVP, leads to QT prolongation without a significant change in repolarization duration [10]. For this reason, the AHA/ACC/HRS recommends use of the JT interval for standardized ECG interpretation in the setting of intraventricular conduction delay [11]. Unlike the QT interval, the JT interval—which reflects only repolarization—is not prolonged during RVP [5, 12], a finding also confirmed in our dataset. However, the JT interval also requires correction for heart rate, typically yielding the JTc interval. Tsai et al. [13] demonstrated excellent correlation between calculated JTc and a simplified derived JTc obtained by subtracting QRS duration from QTc (JTc = QTc—QRS). Yet, since JT and JTc are ultimately derived from QT measurements, accurate QT assessment remains essential for any repolarization evaluation.

Determining the QT interval during RVP is clinically important for understanding arrhythmic risk and managing pacing‐induced alterations in ventricular activation. The QT interval may be influenced not only by RVP but also by the site of stimulation (septal vs. apical), which affects ventricular activation patterns [10]. In our extended model including RV lead position as a covariate, no significant effect was observed.

Several formulas for heart rate correction have been proposed, but none is universally accepted [10, 14, 15, 16]. Bazett's formula remains the most widely used by clinicians and ECG software [17, 18], despite well‐documented limitations and reports of inferiority compared with alternative methods [19, 20]. With RVP as an additional variable, rate correction becomes even more challenging. Our newly derived formula is simple, heart rate‐independent, and demonstrates robust performance in predicting the intrinsic QT interval from paced measurements. It can therefore be considered an optimal method for QT estimation in paced patients.

Previous studies attempted to correct for QRS widening induced by pacing, with mixed results. Bogossian et al. reported that pacing prolongs the QT interval by 48.5% of the paced QRS duration and proposed the formula QTm = QTpaced −0.485 × QRSpaced [5]. While this approach was validated in heart failure patients [7], it systematically overestimated QT by ∼25 ms. In contrast, our formula showed a mean deviation of 15.21 ms in the total population (Figure 5 a+b). Subgroup analysis revealed the smallest deviation in ICD‐female patients (−7.57 ms) and the largest in septal pacing (−21.79 ms). Direct comparison of our formula with the Bogossian method in the same validation cohort demonstrated superior accuracy, with lower mean absolute deviation (26.2 vs. 39.9) and mean squared deviation (1170.5 vs. 2331.6). Both methods tended to overestimate QT at the lower and upper extremes, but our formula produced values more closely aligned with intrinsic QT. Wang et al. [21]also proposed a formula (QTm = QTpaced—(0.86 × QRSpaced −71)), though this was derived from only 22 patients. Chiladakis et al. [4] observed stable pacing‐induced QT prolongation across different heart rates (43 ms in patients with normal baseline QT and 37 ms in those with prolonged QT), suggesting a fixed subtraction method.

**FIGURE 5:**
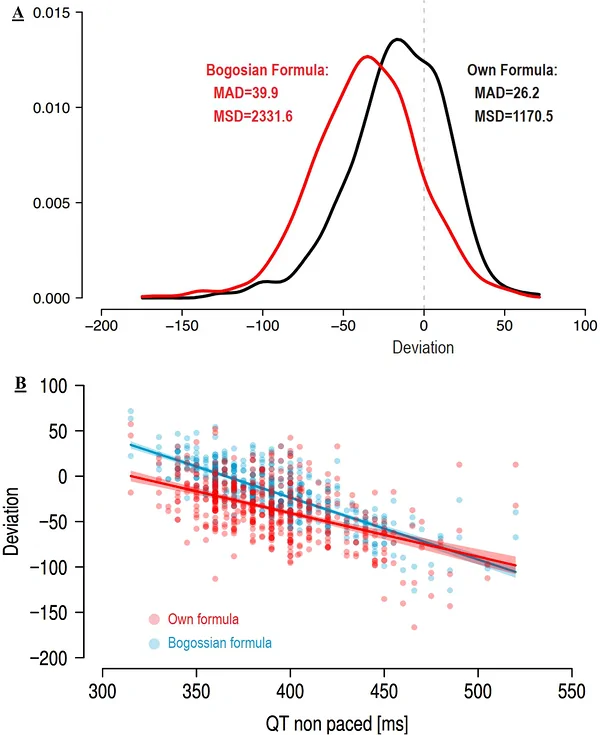
Comparison between our formula and the Bogossian formula, illustrating the mean deviation in the validation groups. A: Density plot. B: Bland‐altman plot, with the observed QT‐nonpaced on the *x* and the deviation (predicted – observed) on the *y*‐axis [Colour figure can be viewed at wileyonlinelibrary.com]

In our cohort, AP at higher heart rates shortened both QT and JT intervals, consistent with intrinsic conduction (∆QT −8.9 ± 18.6 ms, ∆JT −8.8 ± 19.7 ms). By contrast, ventricular pacing prolonged QT while reducing JT (∆QTp +42 ± 28.9 ms, ∆JT −8.6 ± 27 ms).The spline‐based QT correction method, derived from sex‐specific regression models of QT values in a large US database, has been shown to eliminate heart rate dependency and outperform six established formulas. However, its complexity limits clinical applicability compared with simpler approaches.

The present study addresses a long‐standing gap in clinical practice by providing a validated, practical method for QT interval estimation during RVP. Unlike previous methods, our formula is simple, heart rate‐independent, and applicable to a broad patient population. Given that QT monitoring is integral to assessing arrhythmic risk, guiding drug therapy, and ensuring patient safety, the availability of a reliable correction method has direct clinical implications. Importantly, the formula outperformed existing approaches in accuracy, supporting its integration into routine ECG interpretation, particularly in scenarios involving paced patients exposed to QT‐prolonging drugs. By providing a more precise assessment of repolarization in this challenging population, our work contributes to improved patient safety and the standardization of QT interval evaluation in paced rhythms. This study has several important strengths. First, it includes both a dedicated derivation cohort and a large independent validation cohort, thereby strengthening internal validity and reducing the risk of overfitting. Second, QT measurements were obtained under controlled conditions, with matched heart rates, during intrinsic rhythm, AP, and right ventricular pacing (RVP), thereby allowing direct physiological comparison without confounding by heart rate. Third, the study population reflects real‐world device practice, including both pacemaker and ICD recipients and different right ventricular lead positions, supporting broad clinical applicability. Fourth, ECG analysis was performed using standardized high‐resolution digital recordings and independently verified by certified cardiologists, enhancing measurement reliability. Fifth, the proposed formula is simple, heart rate‐independent, and does not require measurement of QRS duration, making it practical for routine clinical use and for potential integration into ECG software. Finally, a direct head‐to‐head comparison with an established correction method in the same validation cohort provides a clear benchmark and strengthens the interpretation of improved agreement.

### 4.1 Limitations

This study has several limitations. First, the proposed formula was not evaluated in patients with cardiac resynchronization therapy (CRT), in whom biventricular activation may influence ventricular repolarization differently. Second, the formula showed a systematic overestimation of intrinsic QT, which may provide a conservative safety margin but could also lead to unnecessary treatment modification. However, the mean deviation was small (−15.21 ms), making clinically relevant misclassification unlikely, although this bias should be considered in practice. Third, patients with complete (100%) ventricular pacing dependency were not included, as preserved intrinsic atrioventricular (AV) conduction was required for direct comparison between paced and non‐paced QT intervals at identical heart rates. Since fully pacing‐dependent patients may exhibit different repolarization characteristics due to chronic pacing‐induced remodeling, the applicability of the formula in this population warrants further dedicated investigation. Finally, only a small proportion of patients were receiving QT‐prolonging drugs, and patients with congenital long QT syndrome or marked drug‐induced QT prolongation were underrepresented; therefore, the performance of the formula in these settings and the potential need for dynamic modification at extreme QT values require further study.

## 5 Conclusion

Identifying QT prolongation in patients with RVP can be challenging, as it requires consideration of drug‐related arrhythmic risk, underlying risk factors, and pacing strategy. In this study, we developed a novel formula that allows the prediction of the QT interval in non‐paced patients based on the QT interval during RVP. The formula is simple to apply, requires only the QT interval measurement, and demonstrates good correlation between predicted and observed QT values. Therefore, it provides a practical tool for estimating the QT interval and assessing arrhythmic risk in patients with VP.

## Funding

The authors have nothing to report.

## Ethics Statement

The study was conducted in accordance with the Declaration of Helsinki. The study included both retrospectively and prospectively enrolled patients. The study protocol was approved by the local ethics committee (No. 327/21).

## Consent

Written informed consent was obtained from prospectively enrolled patients, while the requirement for consent was waived for retrospectively included patients.

## Conflicts of Interest

Amr Abdin received speaker's honoraria from Boston Scientific and Biotronik. Michael Böhm reports personal fees from Abbott, Amgen, Astra Zeneca, Bayer, Boehringer Ingelheim, Cytokinetics, Medtronic, Novartis, ReCor Servier and Vifor. Alhasan Almasri, Yvonne Bewarder, Charles Gieres, Christian Werner and Saarraaken Kulenthiran have no conflict of interests. Christian Ukena received speaker honoraria and consulting fees from Amgen, Bayer, Bristol Myers Squibb, Medtronic, Pfizer, Recor Medical. Christian Werner received speaker's honoraria from Amgen and Daaichi Sankyo.

## Supporting information




**Supporting File 1**: pace70239‐sup‐0001‐SuppMat.docx

## Data Availability

The data underlying this article are available in the article and in its online supplementary material.
