# Supplementary material for: QT Interval Evaluation in Right Ventricular Pacing: Validation of a Novel Formula
Source: Pacing Clin Electrophysiol. 2026 Apr 3;49(10):1455–63. doi: 10.1111/pace.70239 (PMC13638775; doi:10.1111/pace.70239)
Supplement: Supplementary file 1 [file PACE-49-1455-s001.docx]

**QT Interval Measurement in Ventricular Pacing: Validation of a Novel Formula**

**Table S1.** QRS, JT, and QT measurements during atrial pacing (AP) and ventricular pacing (VP) in the derivation cohort.

|  | AP | VP | delta | delta % |
| --- | --- | --- | --- | --- |
| QRS ms | 98,2 | 152,4 | 54,2 | 64,44% |
| JT ms | 276,9 | 275,9 | -1 | 100,36% |
| QT ms | 375,1 | 427,8 | 52,7 | 87,68% |

**Table S2.** QRS, JT, and QT measurements in the non-paced rhythm and during ventricular pacing at 80 bpm (validation cohort)

|  | AP | VP | delta | delta % |
| --- | --- | --- | --- | --- |
| QRS ms | 96,77 | 148,61 | 51,84 | 65,12% |
| JT ms | 293,45 | 289,16 | -4,29 | 101,48% |
| QT ms | 389,93 | 437,56 | 47,63 | 89,11% |

**Table S3.** Mean ± 1.96 SD’ shows the average deviation between predicted and actual non-paced values, with mean ± 1.96 × SD in parentheses. Values are in ms.

|  | **All** | **PM** | **ICD** |
| --- | --- | --- | --- |
| **All** | -15.21 [-73.88, 43.46] | -19.88 [-78.3, 38.54] | -7.6 [-63.7, 48.5] |
| **Male** | -15.52 [-76.81, 45.77] | -21 [-82.51, 40.51] | -8.25 [-66.44, 49.94] |
| **Female** | -14.43 [-66.07, 37.21] | -17.7 [-69.67, 34.27] | -4.8 [-51.21, 41.61] |
| **Apical** | -8.61 [-65.6, 48.38] | -11.68 [-72.73, 49.37] | -7.57 [-63.15, 48.01] |
| **Septal** | -20.88 [-78.8, 37.04] | -21.79 [-79.07, 35.49] | -7.83 [-70.61, 54.95] |

**Figure S1*:*** QRS/QT %. This indicates that QT prolongation during ventricular pacing (VP) is mainly due to QRS widening. AP: atrial pacing

|  | QRS/QT % |
| --- | --- |
| AP | 26.18 |
| VP | 35.62 |

**Figure S2.** Forest plot illustrating the correlation coefficients. The points represent r, and the bars indicate the 95% confidence intervals.

**Figure S3:** A Density plot showing the deviation (Predicted – Measured) on the x-axis for both methods. MAD = Median Absolute Deviation; MSD = Mean Squared Deviation. B: Scatter plot with empirical QT non-paced values on the x-axis and the deviation (Predicted – Measured) on the y-axis.
